# Supplementary material for: The CDK Inhibitor Dinaciclib Improves Cisplatin Response in Nonseminomatous Testicular Cancer: A Preclinical Study
Source: Cells. 2024 Feb 20;13(5):368. doi: 10.3390/cells13050368 (PMC10931172; doi:10.3390/cells13050368)
Supplement: Supplementary file 1 [file cells-13-00368-s001.zip › cells-2798011-supplementary.pdf]

**Supplementary Data**

**Supplementary Methods**

1. **Annexin/PI assay**

The Pacific Blue Annexin V/ SYOX AADVanced apoptosis kit (Invitrogen) was applied as described in Abate et al. Briefly, cells ($5 \times 10^4$ cells/well) were seeded in 6-well plates in complete medium; 24 hours later, cells were treated with the calculated $IC_{50}$ value of CP or dinaciclib for 48 hours. Cells were collected, washed with ice-cold PBS, suspended in the binding buffer, and stained with Pacific Blue Annexin V/ SYOX AADVanced, according to the manufacturer instructions. Cells were then analysed using MACSQuant10 cytometer (Miltenyi) using unlabelled cells as negative control. Quantification of apoptosis was determined by FlowJo v10.6.2 software. Annexin V+/SYTOX- and Annexin V+/SYTOX+ cells were considered early- and late-phase apoptotic cells, according to the manufacturer instructions.

2. **Dinaciclib absorption quantification by liquid chromatography–tandem mass spectrometry (LC-MS/MS)**

Dinaciclib absorption from embryos was evaluated by quantifying the concentration of dinaciclib by liquid chromatography–tandem mass spectrometry (LC-MS/MS). Reagent-grade Acetonitrile for LC-MS and formic acid (98%) were purchased from CARLO ERBA Reagents S.r.l. (Milan, Italy). Ultra-pure water was prepared using a Millipore Milli-Q purification system (Millipore Corporation, Billerica, MA, USA). Dinaciclib and ribociclib standards were purchased from Sigma Aldrich (Sigma Italia, Milano, Italy). Dinaciclib and ribociclib were resuspended in 100 % dimethylsulphoxide (stock solution: 100 mM and 12.5 mM respectively). Drugs were subsequently diluted in methanol, purchased from CARLO ERBA Reagents S.r.l. (Milan, Italy). Ultra performance liquid chromatography (UPLC) was performed using a DionexTM UltiMateTM 3000 Thermo Fisher Scientific S.p.A (Milan, Italy) equipped with a LPG-3400SD quaternary analytical pump, a WPS-3000SL analytical autosampler, a TCC-3000SD thermostatted column compartment. Chromatographic separation was performed using a XSelect CSH C18 column (150 mm × 2.1 mm ID, particle size 3.5 μm) (Waters Corporation, Milano, Italy). The chromatographic system was set up with a flow rate of 0.150 ml/min. The column was kept at 25°C and equilibrated with 100% mobile phase A (water containing 0.1 % formic acid). Mobile phase B (acetonitrile containing 0.1 % formic acid) for the gradient analysis was used. The gradient method was reported in the supplemental Table 1

**Supplemental Table S1**. Gradient method of the ultra-performance liquid chromatography (UPLC) presented as percentage of phase B (acetonitrile) vs time.

| Time (min) | %B |
|---|---|
| 0 | 0 |
| 2 | 0 |
| 5 | 50 |
| 7 | 50 |
| 8 | 100 |
| 10 | 100 |
| 12 | 10 |
| 16 | 10 |
| 17 | 0 |
| 22 | 0 |

The UPLC system was coupled with an electrospray ionization mass spectrometer (LCQ Fleet Ion Trap MSn, Thermo Fisher Scientific). The positive ESI conditions were as follows. The spray voltage was set at 3.60 kV and the source current was set at 100 μA. The capillary voltage was set at 22 V and the capillary temperature was 210 °C. The spray was stabilized with a nitrogen sheath gas (12 arb) and the auxiliary gas was set at 2 arb. The isolation width of precursor ions was 1 mass units. Ions were obtained in the range of m/z 200–800. For all SRM analyses the scan time was equal to 100 ms, the normalized collision energy (NCE) was set to 26 and the isolation width of precursor ions was 2.5 mass units. Data were treated with the Xcalibur software (Version 4.0, Thermo Fisher Scientific). The calibration curves for the quantification of dinaciclib and ribociclib (used as internal standard (IS)) were obtained both as described below. Twenty-five embryos for each batch (up to 120 hpf) were put at 4°C. 100 μl of Internal Standard (IS) (500 nM) was added to each batch with 100 μl of dinaciclib at different dilutions in order to obtain the final concentrations 5-10-25-50-100-200 nM of dinaciclib in methanol. Samples was extracted following the protocol previously reported [30]. Ten μl of each sample were analyzed by LC-MS/MS (LOD 1 nM; LOQ 2.5 nM). The SRM quantifier transitions were m/z 397.4- ->379.4 for dinaciclib and m/z 435.3-->322.3 for ribociclib.

**Supplemental Table S2.** Primary antibodies.

| Target | Code | Species | Dilution | Company |
|---|---|---|---|---|
| CDK1 | ab18 | Mouse | 1:500 | Abcam (Cambridge, UK) |
| CDK2 (D-12) | sc-6248 | Mouse | 1:250 | Santa Cruz Biotechnology (Dallas, TX, USA) |
| CDK5 (J-3) | sc-6247 | Mouse | 1:200 | Santa Cruz Biotechnology |
| CDK9 (D-7) | sc-13130 | Mouse | 1:200 | Santa Cruz Biotechnology |
| Ciclina I | PA-5-66397 | Rabbit | 1:1000 | Invitrogen (Milan, Italy) |
| Ciclina E (E-4) | sc-377100 | Mouse | 1:200 | Santa Cruz Biotechnology |
| Ciclina A (B-8) | sc-271682 | Mouse | 1:200 | Santa Cruz Biotechnology |
| Ciclina K (G-11) | sc-376371 | Mouse | 1:100 | Santa Cruz Biotechnology |
| Ciclina T1 (E-3) | sc-271348 | Mouse | 1:250 | Santa Cruz Biotechnology |
| Ciclina B2 (A-2) | sc-28303 | Mouse | 1:125 | Santa Cruz Biotechnology |
| β-tubulina | T2200-200UL | Rabbit | 1:2000 | Sigma-Aldrich (Milan, Italy) |

**Supplemental Table S3.** Sequences of oligonucleotide primers for qRT-PCR

| | | Nucleotide Sequence | Product size (bp) |
|---|---|---|---|
| p35 | F | CAGATCCAAGGGGGCAGC | 86 |
| | R | AGGGATAAAACCGCTCACCG | |
| p39 | F | CCCCGTAGACCCCTGTTTCT | 75 |
| | R | GGTGACACATGACAGGAGCAA | |
| Cyclin I | F | TACAGCAAGCTGGGTAGCAG | 134 |
| | R | CCGCACATTCACTTTCCACA | |
| β-actin | F | TCTTCCAGCCTTCCTTCCTG | 146 |
| | R | CAATGCCAGGGTACATGGTG | |

**Supplemental Table S4**. Gene expression of p35, p39 and Cyclin I.

| **Cell model** | **ΔCt ± SD** | | |
|---|---|---|---|
| | **p35** | **p39** | **Cyclin I** |
| NT2/D1 | 17.5 ± 2.1 | 17.3 ± 1.7 | 5.6 ± 0.5 |
| NT2/D1 - R | 19 ± 0.8 | 13 ± 1.4 | 6.1 ± 0.9 |
| NCCIT | 19.2 ± 0.5 | 14.9 ± 0.9 | 5.9 ± 0.7 |
| NCCIT - R | 19 ± 0.1 | 12.7 ± 0.9 | 5.8 ± 0.3 |

**Supplementary Figures**

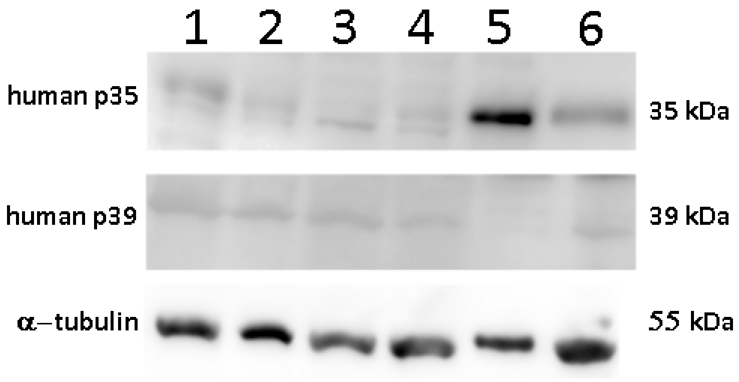


**Figure S1.** p35 and p39 protein expression in non-seminoma cell models. 100 µg of non-seminoma protein lysate were separated with a 4-12% Bis-Tris gel as described in methods. Lane 1: NT2/D1, lane 2: NT2/D1-R, lane 3: NCCIT, lane 4: NCCIT-R, lane 5: rat hippocampus, lane 6: rat midbrain. Rat hippocampus and midbrain were used as positive control.

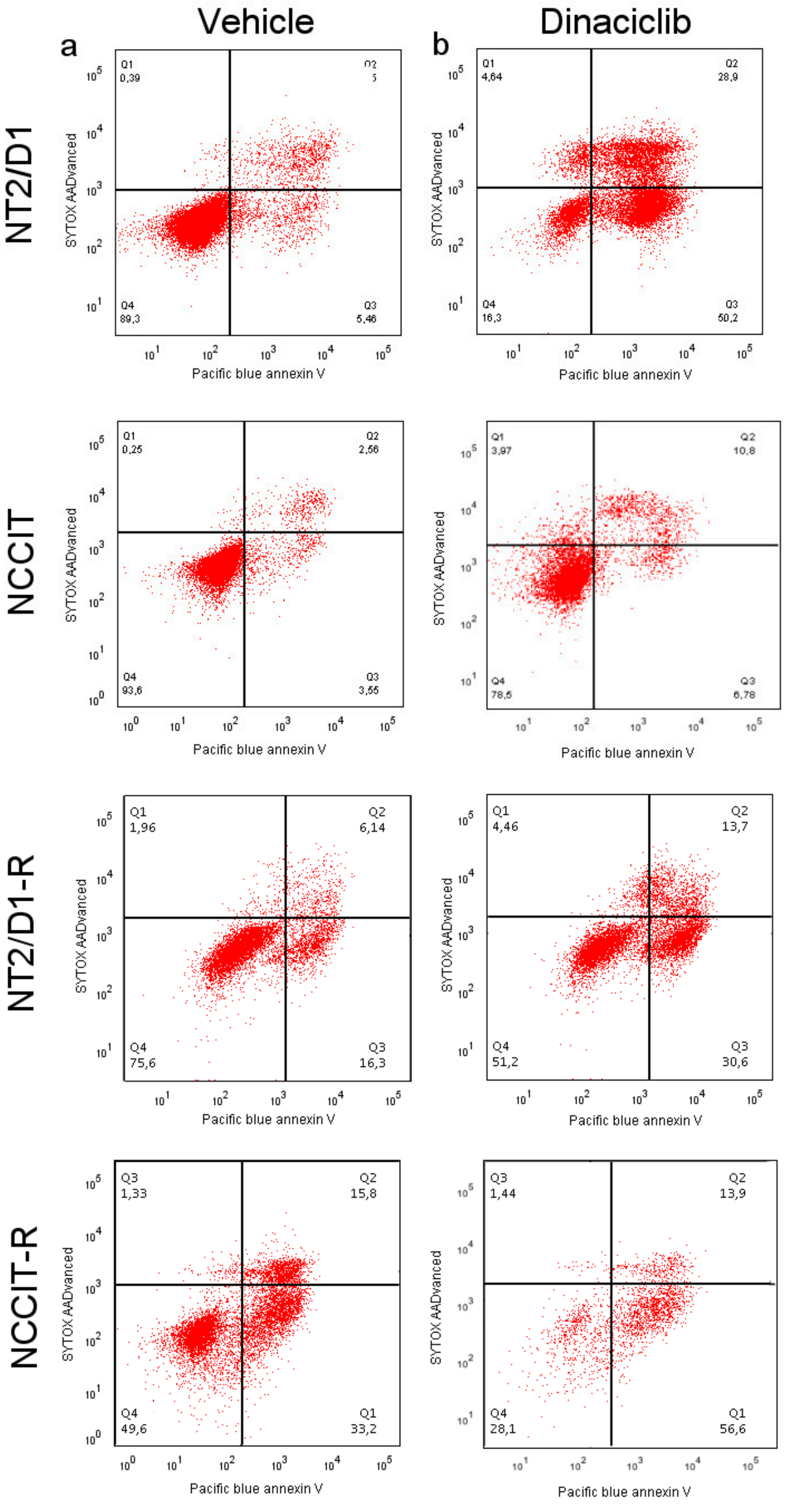


**Figure S2.** Representative images of NT2/D1 , NCCIT, NT2/D1-R and NCCIT-R cell apoptosis in untreated cells (a) or after 48 hours' treatment with dinaciclib (b). Alive cells are shown in the lower left part of the panel (Q4); early apoptotic cells are shown in the lower right part of the panel (Q3); late apoptotic cells are shown in the upper right part of the panel (Q2); necrotic cells are shown in the upper left part of the panel (Q1).

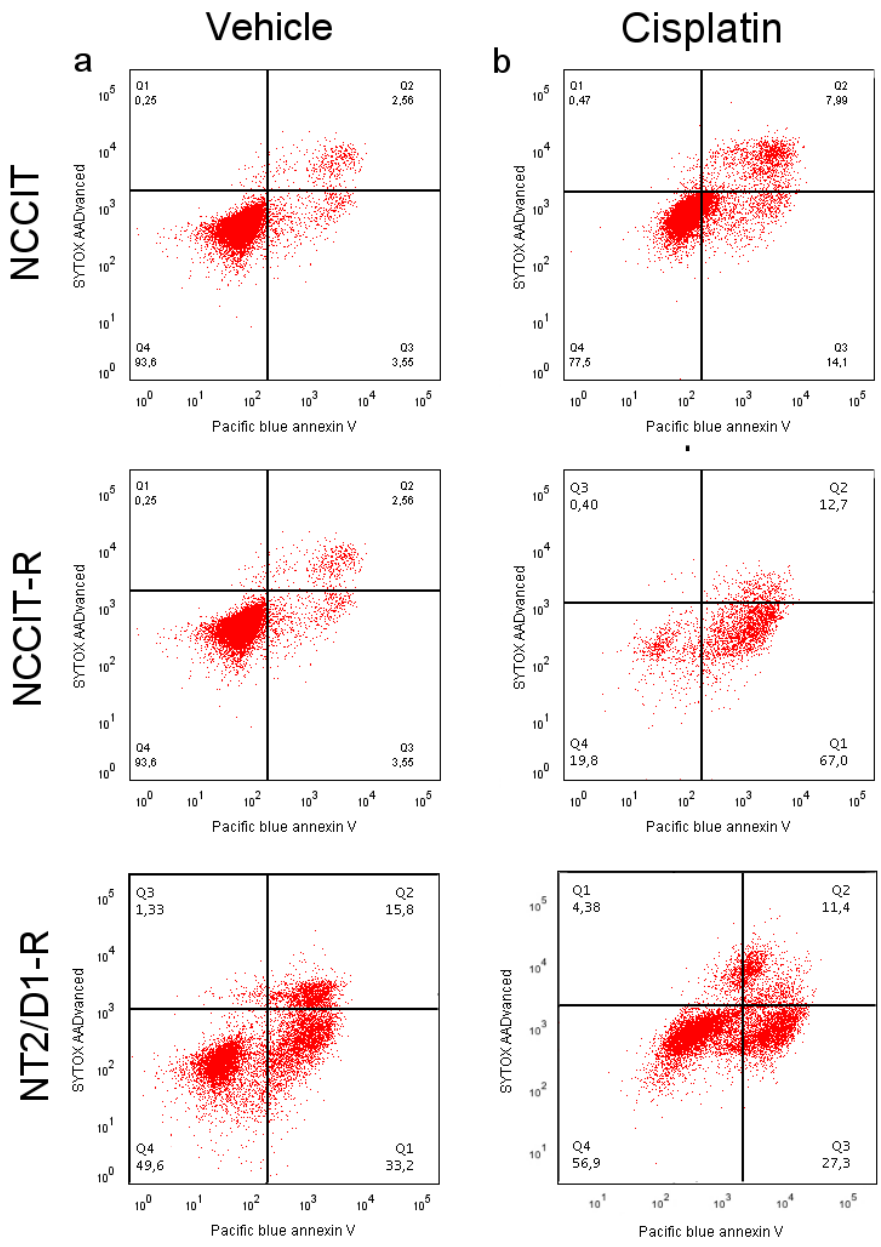


**Figure S3.** Representative images of NCCIT, NCCIT-R and NT2/D1 cell apoptosis in untreated cells (a) or after 48 hours' treatment with CP (b). The apoptotic effect of cisplatin on NT2/D1 cells was previously demonstrated in Rossini et al., 2021 [17]. Alive cells are shown in the lower left part of the panel (Q4); early apoptotic cells are shown in the lower right part of the panel (Q3); late apoptotic cells are shown in the upper right part of the panel (Q2); necrotic cells are shown in the upper left part of the panel (Q1).

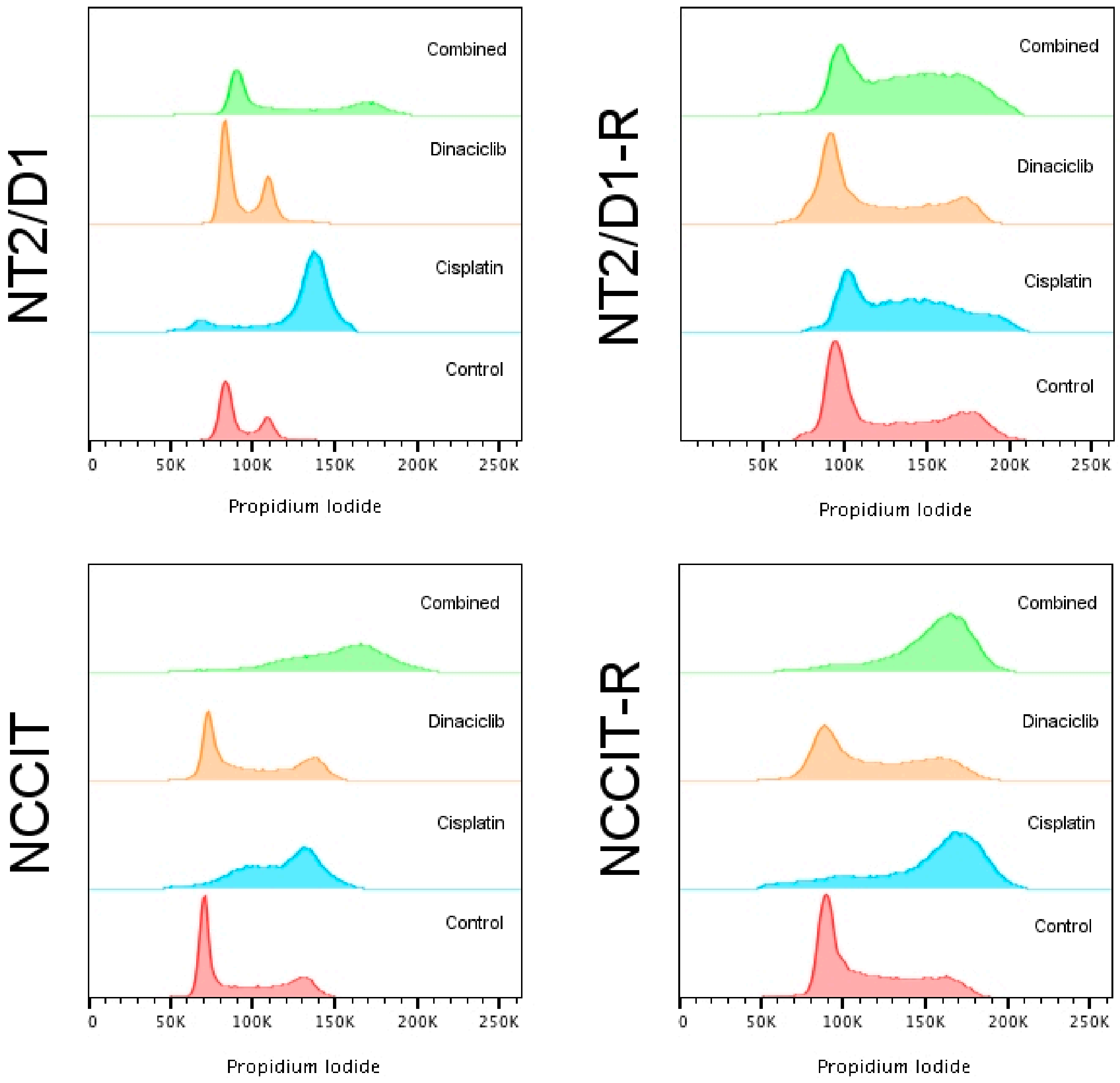


**Figure S4.** Cell-cycle analysis after dinaciclib, cisplatin and their combination treatment in NT2/D1, NT2/D1-R, NCCIT and NCCIT-R cells.

*Supplemental results*

*Effect of dinaciclib alone or combined with cisplatin in the Zebrafish/tumor xenograft model*

For in vivo experiments, the area values were modeled on a logarithmic scale using an ordinary least squares (OLS) linear model, which is the accepted model for evaluating the effects of drugs and their interactions. The inclusion of interaction terms allows us to estimate the effect of jointly adding the two drugs, enabling an evaluation of whether the combination yields an effect that deviates from the simple additive effects of the individual drugs, broadly described as a synergistic or antagonistic effect.

The results are presented in Supplemental Table 5 as estimates, represented by the ratio between group mean values, along with their corresponding 95% confidence intervals. Cis*Din Ixn represents the excess (or

deficiency) effect compared to the simple additive effect, indicating synergy. If the ratio is greater than 1, it means that the reduction compared to the carrier of the addition of Cis+Din is lower than the pure additive effect. Cis+ denotes the addition of Cisplatin only, while Din+ denotes the addition of Dinaciclib only. Cis*Din Ixn indicates the interaction between Cisplatin and Dinaciclib.

Supplemental Table 5. Interaction between cisplatin and dinaciclib in zebrafish embryos xenografts.

| **Cell line** | **Contrast** | **Ratio (95% CI)** | **p.value** |
|---|---|---|---|
| NCCIT | Cis+ | 0.77 (0.64 - 0.93) | <0.01 |
| | Din+ | 0.81 (0.68 - 0.97) | 0.023 |
| | Cis*Din Ixn | 1.07 (0.82 - 1.38) | 0.632 |
| NCCIT-R | Cis+ | 0.86 (0.76 - 0.99) | 0.035 |
| | Din+ | 0.63 (0.55 - 0.71) | <0.01 |
| | Cis*Din Ixn | 1.10 (0.91 - 1.31) | 0.324 |
| NT2/D1 | Cis+ | 0.71 (0.63 - 0.81) | <0.01 |
| | Din+ | 0.60 (0.53 - 0.68) | <0.01 |
| | Cis*Din Ixn | 1.32 (1.10 - 1.58) | <0.01 |
| NT2/D1-R | Cis+ | 0.84 (0.73 - 0.98) | 0.030 |
| | Din+ | 0.70 (0.61 - 0.80) | <0.01 |
| | Cis*Din Ixn | 0.92 (0.75 - 1.13) | 0.410 |

## References


17. Rossini, E.; Bosatta, V.; Abate, A.; Fragni, M.; Salvi, V.; Basnet, R.M.; Zizioli, D.; Bosisio, D.; Piovani, G.; Valcamonico, F.; et al. Cisplatin Cytotoxicity in Human Testicular Germ Cell Tumor Cell Lines Is Enhanced by the CDK4/6 Inhibitor Palbociclib. *Clin. Genitourin. Cancer* **2021**, *19*, 316–324. https://doi.org/10.1016/j.clgc.2021.01.006.
30. Gianoncelli, A.; Guarienti, M.; Fragni, M.; Bertuzzi, M.; Rossini, E.; Abate, A.; Basnet, R.M.; Zizioli, D.; Bono, F.; Terzolo, M.; et al. Adrenocortical Carcinoma Xenograft in Zebrafish Embryos as a Model To Study the In Vivo Cytotoxicity of Abiraterone Acetate. *Endocrinology* **2019**, *160*, 2620–2629. https://doi.org/10.1210/en.2019-00152.
